# Supplementary material for: Evaluation of the Implementation of the Dutch Breast Cancer Surveillance Decision Aid including Personalized Risk Estimates in the SHOUT-BC Study: A Mixed Methods Approach
Source: Cancers (Basel). 2024 Mar 31;16(7):1390. doi: 10.3390/cancers16071390 (PMC11010914; doi:10.3390/cancers16071390)
Supplement: Supplementary file 1 [file cancers-16-01390-s001.zip › cancers-2924960-supplementary.pdf]

Supplementary materials

Supplementary Figure S1 **Participant flowchart**

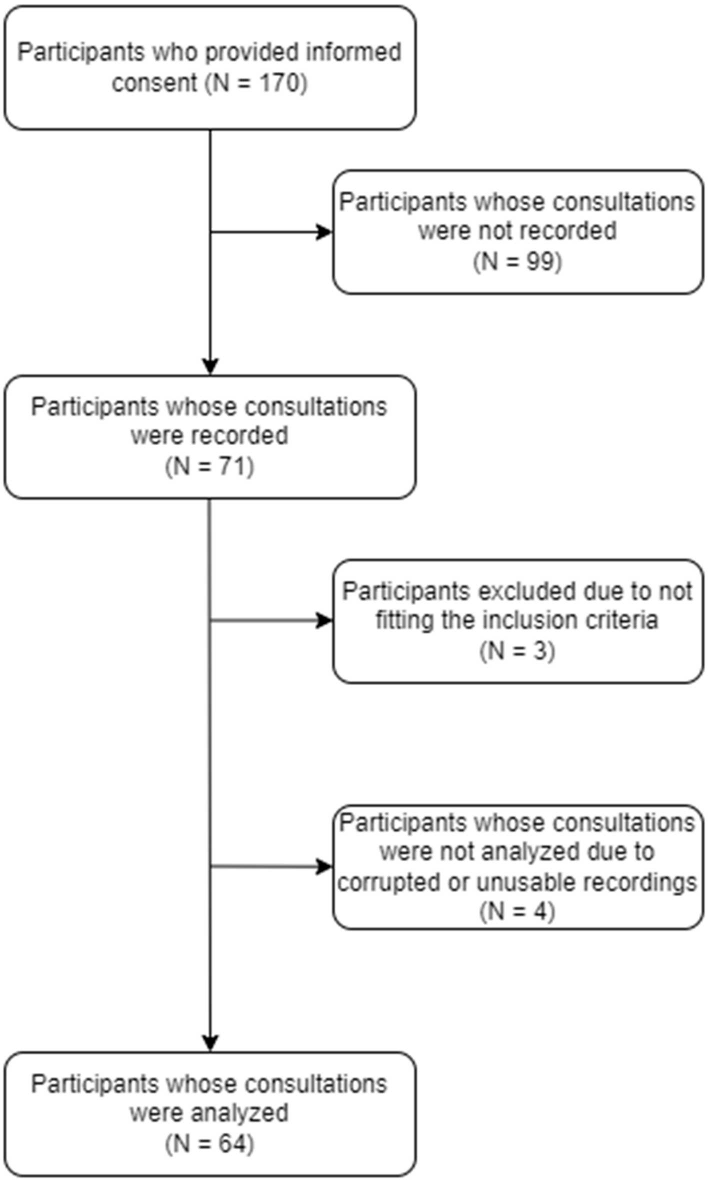

Supplementary Table S1. Determinants for use of the BCS-PtDA by HCPs as measured with the MIDI (N= 24)

**Determinants associated with the innovation (BCS-PtDA)**			
	**Totally agree/ Agree (%)**	**Neutral (%)**	**Totally disagree/ Disagree (%)**
**Procedural clarity:** it is clear which activities I should perform when offering the BCS-PtDA	79	8	13
**Correctness:** the BCS-PtDA is based on factually correct knowledge	88	4	8
**Completeness:** all information and materials to work with the BCS-PtDA properly are provided	83	4	13
**Complexity:** the BCS-PtDA is not too complex for me to use[a]	83	13	4
**Compatibility:** the BCS-PtDA is a good match for how I am used to working	58	25	17
**Observability:** the outcomes of using the BCS-PtDA are clearly observable	29	33	38
**Relevance for patient:** the BCS-PtDA is relevant for my patients	58	33	8
**Determinants associated with the users (i.e., patients and HCPs)**			
	**Totally agree/ Agree (%)**	**Neutral (%)**	**Totally disagree/ Disagree (%)**
**Personal benefits:** the BCS-PtDA saves me time in informing my patients	0	29	71
**Personal benefits:** the BCS-PtDA provides me more time to discuss the considerations and preferences of my patients with them	25	25	51
**Personal drawback:** my workload has not increased by using the BCS-PtDA[a]	8	25	67
**Outcome expectations (importance):** the BCS-PtDA helps to create awareness that there is a choice regarding the organization of post-treatment surveillance	92	4	4
**Outcome expectations (importance):** the BCS-PtDA helps to inform about and discuss the different options regarding the organization of post-treatment surveillance	79	13	8
**Outcome expectations (importance):** the BCS-PtDA helps to clarify the wishes and preferences of my patients regarding the organization of post-treatment surveillance	75	17	8
**Outcome expectations (importance):** the BCS-PtDA helps to make a shared decision regarding the organization of post-treatment surveillance	79	17	4
	**Most definitely/ definitely (%)**	**Neutral (%)**	**Most definitely not/ definitely not (%)**
**Outcome expectations (probability):** the BCS-PtDA helps to create awareness that there is a choice regarding the organization of post-treatment surveillance	71	25	4
**Outcome expectations (probability):** the BCS-PtDA helps to inform about and discuss the different options regarding the organization of post-treatment surveillance	75	25	0
**Outcome expectations (probability):** the BCS-PtDA helps to clarify the wishes and preferences of my patients regarding the organization of post-treatment surveillance	54	42	4
**Outcome expectations (probability):** the BCS-PtDA helps to make a shared decision regarding the organization of post-treatment surveillance	58	42	0

Abbreviations: *HCP* health care professional, *BCS-PtDA* Breast Cancer Surveillance Decision Aid, *PROM* patient-reported outcome measure.
[a] Determinant is reversed for readability/interpretability.
[b] Answer categories were divided into: 1) “a majority, almost all colleagues, all colleagues”; 2) “half of colleagues”; and 3) “not a single colleague, almost no colleague, a minority”.
[c] Possible answer categories were divided into: “true” (= facilitator) and “false” (= barrier); answering option: “I don’t know” not considered as part of categorization as facilitator or barrier.

Supplementary Table 1 continued Determinants for use of the BCS-PtDA by HCPs as measured with the MIDI (N= 24)

**Determinants associated with the users (i.e., patients and HCPs)**			
	**Totally agree/ Agree (%)**	**Neutral (%)**	**Totally disagree/ Disagree (%)**
**Professional obligation:** I feel that it is my responsibility to use the BCS-PtDA	63	21	17
**Patient satisfaction:** patients are generally satisfied when I use the BCS-PtDA	54	29	17
**Patient cooperation:** patients generally cooperate when I use the BCS-PtDA	58	33	8
**Social support:** I can count on adequate assistance from my colleagues if I need it to use the BCS-PtDA	83	17	0
**Social support:** I can count on adequate assistance from my superior if I need it to use the BCS-PtDA	54	46	25
	**A majority, almost all colleagues, all colleagues (%)**	**Half of colleagues (%)**	**Not a single colleague, almost no colleague, a minority (%)**
**Descriptive norm:** proportion of colleagues that actually use the BCS-PtDA[b]	67	17	17
	**Most definitely/ definitely (%)**	**Neutral (%)**	**Most definitely not/ definitely not (%)**
**Normative beliefs:** my patients expect me to work with the BCS-PtDA	8	46	46
**Normative beliefs:** my colleagues expect me to work with the BCS-PtDA	67	21	13
**Normative beliefs:** my superior expects me to work with the BCS-PtDA	33	50	17
**Normative beliefs:** the Board of Directors of my hospital expects me to work with the BCS-PtDA	29	50	21
**Normative beliefs:** my colleagues from other Santeon hospitals expect me to work with the BCS-PtDA	54	46	0
**Motivation to comply:** I find the opinion of my patients important when it comes to working with the BCS-PtDA	96	4	0
**Motivation to comply:** I find the opinion of my colleagues important when it comes to working with the BCS-PtDA	75	25	0
**Motivation to comply:** I find the opinion of my superior important when it comes to working with the BCS-PtDA	25	25	50
**Motivation to comply:** I find the opinion of the Board of Directors of my hospital important when it comes to working with the BCS-PtDA	29	33	38
**Motivation to comply:** I find the opinion of my colleagues from other Santeon Hospitals important when it comes to working with the BCS-PtDA	63	17	21
**Self-efficacy:** I am able to calculate the personal risks for recurrence of breast cancer using the INFLUENCE 2.0-nomogram	92	8	0
**Self-efficacy:** I am able to discuss the personal risks for recurrence of breast cancer with patients	92	8	0
**Self-efficacy:** I am able to fill in the consultation sheet	92	4	4
**Self-efficacy:** I am able to explain the consultation sheet	96	4	0
**Self-efficacy:** I am able to refer to the online BCS-PtDA	88	4	8
**Self-efficacy:** I am able to explain the online BCS-PtDA	83	13	4
**Self-efficacy:** I am able to discuss the PROM score on fear of cancer recurrence with patients	50	29	21
**Self-efficacy:** I am able to discuss the summary sheet in consultation with patients	75	17	8

Abbreviations: *HCP* health care professional, *BCS-PtDA* Breast Cancer Surveillance Decision Aid, *PROM* patient-reported outcome measure.
MIDI determinants that were answered by ≥20% of HCPs with "totally disagree/disagree" or "most definitely not/ definitely not" were considered barriers, and items answered by ≥80% with "agree/totally agree" or "most definitely/definitely" were considered facilitators.
[a] Determinant is reversed for readability/interpretability.
[b] Answer categories were divided into: 1) "a majority, almost all colleagues, all colleagues"; 2) "half of colleagues"; and 3) "not a single colleague, almost no colleague, a minority".
[c] Possible answer categories were divided into: "true" (= facilitator) and "false" (= barrier); answering option: "I don't know" not considered as part of categorization as facilitator or barrier.

Supplementary Table 1 continued Determinants for use of the BCS-PtDA by HCPs as measured with the MIDI (N= 24)

**Determinants associated with the organization (i.e., hospital)**			
	**Totally agree/ Agree (%)**	**Neutral (%)**	**Totally disagree/ Disagree (%)**
**Time available:** there is enough time available to integrate the BCS-PtDA as intended in my day-to-day work	25	17	58
**Material resources and facilities:** there are enough materials and facilities provided to use the BCS-PtDA as intended	71	17	13
**Information accessible:** it is easy for me to find information about using the BCS-PtDA as intended	75	17	8
**Performance feedback:** feedback is regularly provided about progress with the implementation of the BCS-PtDA	50	21	29
	**True**		**False**
**Formal ratification by management:** there are formal arrangements relating the use of the BCS-PtDA[c]	33		33
**Coordinator:** one or more people have been designated to coordinate the process of implementing the BCS-PtDA[c]	88		4
**Unsettled organization:** there aren't any other changes going on that could influence implementation of the BCS-PtDA [a,c]	25		58

Abbreviations: *HCP* health care professional, *BCS-PtDA* Breast Cancer Surveillance Decision Aid, *PROM* patient-reported outcome measure.
[a] Determinant is reversed for readability/interpretability.
[b] Answer categories were divided into: 1) "a majority, almost all colleagues, all colleagues"; 2) "half of colleagues"; and 3) "not a single colleague, almost no colleague, a minority".
[c] Possible answer categories were divided into: "true" (= facilitator) and "false" (= barrier); answering option: "I don't know" not considered as part of categorization as facilitator or barrier.
